# Model of the Performance Based on Artificial Intelligence–Fuzzy Logic Description of Physical Activity

**DOI:** 10.3390/s23031117

**Published:** 2023-01-18

**Authors:** Adam Szulc, Piotr Prokopowicz, Krzysztof Buśko, Dariusz Mikołajewski

**Affiliations:** 1 Institute of Physical Education, Kazimierz Wielki University in Bydgoszcz, 85-064 Bydgoszcz, Poland; 2 Institute of Computer Science, Kazimierz Wielki University in Bydgoszcz, 85-064 Bydgoszcz, Poland

**Keywords:** posture-movement assessment, deaf athletes, computational models, fuzzy logic

## Abstract

**Featured Application:**

**Potential applications of the work are systems for objective artificial-intelligent performance assessment in healthy individuals (including athletes) and patients with various injuries and conditions, including within the eHealth paradigm, including future wearable devices (e.g., e-shoes).**

**Abstract:**

The aim of the study was to build a fuzzy model of lower limb peak torque in an isokinetic mode. The study involved 93 male participants (28 male deaf soccer players, 19 hearing soccer players and 46 deaf untraining male). A fuzzy computational model of different levels of physical activity with a focus on the lower limbs was constructed. The proposed fuzzy model assessing lower limb peak torque in an isokinetic mode demonstrated its effectiveness. The novelty of our research lies in the use of hierarchical fuzzy logic to extract computational rules from data provided explicitly and then to determine the corresponding physiological and pathological mechanisms. The contribution of our research lies in complementing the methods for describing physiology, pathology and rehabilitation with fuzzy parameters, including the so-called dynamic norm embedded in the model.

## 1. Introduction

The monitoring and early diagnosis of motor skills and cognitive performance in both healthy and sick people [1,2], including deaf athletes plays an important role [3,4]. The complexity of the mechanisms and the large number of parameters that need to be taken into account make artificial intelligence(AI) a great potential solution for speeding up and increasing the accuracy of such analyses. Performance analysis based on artificial intelligence is increasingly being used, both to assess motor abilities [5,6,7], and cognitive abilities [8,9] allowing not only faster extraction of mechanisms and relationships between data, but also proving itself in the area of uncertain, incomplete data or data from non-homogeneous groups. Hence the validity and novelty of the present study, providing prototype fuzzy models.

Current research was carefully reviewed, key publications identified, including most controversial and diverging hypotheses [10,11,12,13]. The mechanisms that stabilize posture (stretch reflexes, intermuscular reflexes and intrinsic muscle properties) resist deviation from posture, in which the balance of muscular and external forces is maintained. For each muscle, these mechanisms become functional at a certain threshold (expressed in muscle length, corresponding joint angle). Furthermore, spinal and supraspinal centers can shift the above spatial thresholds for individual muscle groups. The control strategy described in this way allows us to conclude that postural and movement stability is provided by a common mechanism [10].

Damage to the postural control system can cause various context-specific instabilities. Computational modeling of the aforementioned mechanisms supports the understanding of multiple postural controls and supports effective balance rehabilitation to improve mobility and prevent falls [14,15,16].

Fuzzy logic-based approaches to physical activity assessment are not common, although some progress has already been made [17]. To date, fuzzy logic has mainly been used for pattern recognition in medical signals [18], especially with linguistic description, uncertainty and incompleteness of data. A review of the current state of knowledge shows that fuzzy logic has so far been used to diagnose diseases based on symptoms, and historical and clinical data, including among others the fields of celiac disease, diabetes, iris, heart, breast cancer, dental, cholera, brain tumor, liver, asthma, viral, Parkinson’s disease, lung, kidney, Huntington’s disease and chest diseases [19,20,21,22,23,24,25]. The benefits of fuzzy logic are expected primarily in injuries, conditions and deficits that have not found algorithms to detect them early and accurately and need artificial intelligence support to do so [20]. Agent-based modeling and fuzzy logic allow the simulation of the experience of a human expert, are simple to set up and provide high-level functions (connections and decision-making processes) [26]. Ordered Fuzzy Numbers (OFN) can also be used for modeling changes in dynamic processes [27,28].

There is still a lack of simpler models that are easy to apply clinically, not only in sick people, but also in healthy people, including those actively involved in sport.

The aim of study was to build a fuzzy model of lower limb peak torque in an isokinetic mode.

## 2. Materials and Methods

### 2.1. Material

The study involved 93 male participants (28 male deaf soccer players, 19 hearing soccer players and 46 deaf untrained males) (Table 1). The method of selection was convenience sampling.

### 2.2. Methods

Biodex System 4 Pro—a set for neuromuscular assessment and training in an isokinetic mode (eccentric and concentric) was used to assess of peak torque of lower limb the study participants. The isokinetics test is used to check the ability of patients to generate peak torque of the lower limb in an isokinetic mode with different angular velocities (60 deg/s, 180 deg/s, 300 deg/s).

The balance between the left leg and right leg and between quadriceps and hamstring muscles of left and right leg is very important.

The weaker the muscles, the poorer the stability and therefore the greater the risk of injury. The main characteristics of the Biodex System 4 Pro are:

- Easy and precise alignment of the dynamometer’s axis of motion with that of the joint being tested/exercised;
- Repeatability of the dynamometer setting;
- Force torque measurement during acceleration and deceleration phases of movement;
- Fully adjustable adjustment for assessment and training of different joints and muscle groups;
- Torque measurement range: concentric operation from 0 to 680 Nm, eccentric operation from 0 to 544 Nm;
- Range of motion speed measurement: concentric operation up to 500 deg/s, eccentric operation up to 300 deg/s;
- Minimum movement speed (for passive movement): from 0.25 deg/s;
- Minimum force torque (for passive movement): 0.68 Nm and above;
- Minimum force torque value (for isotonic operation): 0.68 Nm and above.

The Biodex System 4 Pro is used in the training and rehabilitation of orthopedic patients. For people who have undergone surgery (especially lower limb surgery) or trauma, it allows a quick and safe return to full physical fitness. It can also be used as a sensomotor training device. Thanks to the use of visualization, it enables biofeedback in real time, allowing faster progress in the exercise. The system also makes it possible to assess whether the subject is at risk of injury (e.g., to the knee joint) and possibly suggest preventive exercises to minimize the risk of injury (e.g., during a ski trip).

Each study subject participated in a lower limb assessment on the Biodex stand (five repetitions away and towards). Defined as:

- Away (extension)—movement of the lower limb at the knee joint in the sagittal plane between 90 and 0 degrees—as straightening;
- Toward (flexion)—(movement in the opposite direction to away) as bending after previous straightening.

Three parameters were assessed at three speeds of movement in the isokinetic mode of the limited lower limb: 60 deg/s, 180 deg/s and 300 deg/s angular velocity:

- PEAKTQ (R, L)—peak torque value (R—lower limb right, L—lower limb left) (Nm);
- PEAKTQ/BM—the ratio of peak torque to body mass (Nm/kg);
- H/Q—the ratio of peak torque of hamstring to quadriceps (flexors to extensors of lower limb) (%);

### 2.3. Statistical Analysis

Data were stored in an MS Excel spreadsheet (Microsoft, Redmond, WA, USA), and Statistica 13 software (StatSoft, Tulsa, OK, USA) was used for statistical analysis. Data distribution was checked using the Shapiro–Wilk test. For data with a near-normal distribution, description using a mean and SD (standard deviation)and parametric tests were used. For data with a distribution different from normal, Min—minimum, Q1—lower quartile, median, Q3—upper quartile, max—maximum values and non-parametric tests were used. Correlations among values from measurements and outcomes from the model were calculated by non-parametric Spearman’s test.

The threshold for statistical significance was set at *p* ≤ 0.05.

### 2.4. Computational Analysis

The researcher’s own software for fuzzy systems/ordered fuzzy numbers (including fuzzy logic libraries built by Piotr Prokopowicz)was used to build the computational models.

Based on the gathered data sets, a fuzzy computational model of different levels of physical activity, with a focus on the lower limbs, was constructed. The purpose of a fuzzy system (FS) is to provide rules or to model expected sets of parameter relationships that can be derived from knowledge (expert knowledge of the field), research assumptions (physical/medical formulas/relationships), or from measurements made on a reference group.

In choosing a hierarchical fuzzy model in this case, we considered two alternatives (large systems involving 3–4 levels of hierarchy):

- Two evaluation systems, including one with detailed data (both legs), and another simpler one where only the data for the leading leg were considered to see if the results were similar;
- A comparative evaluation between hearing players and deaf players—more than one fuzzy system to indicate a better structure of the algorithm.

In this article, standard trapezoidal fuzzy sets (fuzzy intervals) are mainly used. For their description, a notation similar to the description of LR fuzzy sets is used, but the order of the values is changed.

Following that, a trapezoidal fuzzy set T may be described as:

*T* = (*l*, *k*_1_, *k*_2_, *r*)

where:

*l*, *r*—left (low) and right (maximum) boundaries of the support of *T*; *k*_1_,*k*_2_ represents the kernel interval of *T*.

This description of the fuzzy set is consistent with the standard adopted in many popular scientific tools (Matlab: Fuzzy Logic Toolbox*,* Octave: fuzzy logic toolkit package, Scilab: sciFLT—Fuzzy Logic Toolbox, etc.).

For the research in this paper, the six data inputs (gathered from Biodex) were separated for each isokinetic mode. The first two consist of the module for the evaluation of maximal peak torque. They are:

- R AGON/ANTAG (denoted in fuzzy system by *X_R_^MT^*)—the ratio of extensor to flexor muscles for a right leg,
- L AGON/ANTAG*(X_L_^MT^*)—as per previous, but for a left leg.

The next module—relative torque estimation—is based on four other parameters from the Biodex Balance System. They are:

- R PEAKTQ/BM AWY(*X_RAWY_^RT^*)—peak torque relative to body mass for a right leg during away move,
- L PEAKTQ/BM AWY (*X_LAWY_^RT^*)—as per previous, but for a left leg,
- R PEAKTQ/BM TWD (*X_RTWD_^RT^*)—peak torque relative to body mass for a right leg during toward move,
- L PEAKTQ/BM TWD (*X_LTWD_^RT^*)—as per previous, but for a left leg.

The intervals of values for each of above parameters were divided for each isokinetic mode, and are presented in Table 2. In that table the basic linguistic interpretation is also provided. This information was used as source for the definition of the fuzzy system boundaries.

**Figure 1:**
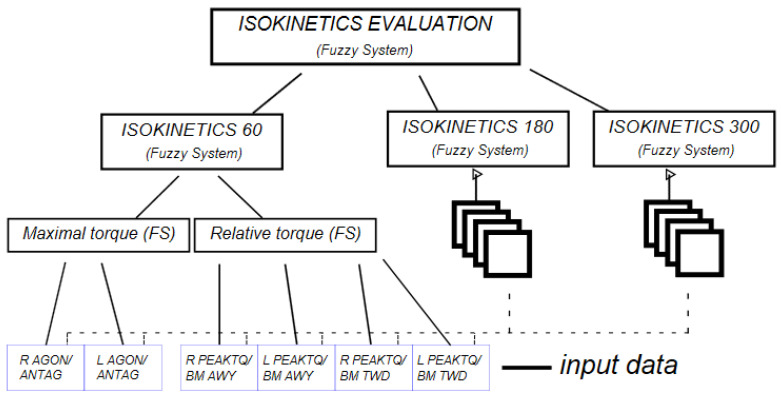
The structure of the hierarchy of fuzzy system.

In general, the proposed hierarchical system for the evaluation the isokinetics of lower body parts of humans can be divided into three separate parts. Each is related to another isokinetic mode in Biodex. Furthermore, each isokinetic mode is divided into two modules: the module for the evaluation of the maximal peak torque (maximal torque on Figure 1) and the module for the evaluation of torque relative to body mass (relative torque in Figure 1).

The resulting fuzzy system is shown in Figure 1.

The structure of the model is a three-level hierarchical system divided into three modules: Isokinetics 60, Isokinetics 180 and Isokinetics 300. All these parts together make up the whole isokinetics evaluation.

It is important to note that although these isokinetics partial modules have a similar structure, they use different intervals/scopes, which are presented in Table 2.

The input data for each isokinetics mode consist of six components. Here, we present thee description for the Isokinetics 60 mode. The other modes of the fuzzy systems are defined in similar way based on the related data presented in Table 2.

The first—lower—tier comes from two modules, the module of maximal torque *X^MT^* on and the relative torque *X^RT^* (see Figure 1).

Input data for Isokinetics 60:

*X^MT^* = {*X_R_^MT^*, *X_L_^MT^*}, *X^RT^* = {*X_RAWY_^RT^*, *X_LAWY_^RT^*, *X_RTWD_^RT^*, *X_LTWD_^RT^*},

where each of them is divided into fuzzy sets as follows (described as fours, in a trapezoid shape):

*X_R_^MT^: ^low^X_R_^MT^ =* (0;0;0;45),*^med^X_R_^MT^ =* (0;45,65,100),*^high^X_R_^MT^ =* (65;100;100;100), *X_RAWY_^RT^*: *^low^X_RAWY_^RT^* = (0;0;0.6;3.4), *^high^X_RAWY_^RT^* = (0.6;3.4;4.5;4.5), *X_RTWD_^RT^*: *^low^X_RTWD_^RT^* = (0;0;0.4;2.2), *^high^X_RTWD_^RT^* = (0.4;2.2;3;3). (1)

The fuzzy sets boundaries presented above are for the right leg, and are the same as for the left leg.

For the output, we use the following variables:

Maximal torque module:

*Y^MT^ =* {*Y*_1_; *Y*_2_; *Y*_3_}: *Y*_1_ = (0; 0; 0; 0.5), *Y*_2_ = (0; 0.5; 0.5; 1), *Y*_3_ = (0.5; 1; 1; 1).

Relative torque module:

*Y^RT^ =* (*Y*_1_*;Y*_2_*;Y*_3_;*Y_4_*;*Y*_5_)*: Y*_1_
*=* (0;0;0;0.25), *Y*_2_
*=* (0;0.25;0.25;0.5), *Y*_1_
*=* (0.25;0.5;0.5;0.75), *Y*_4_
*=* (0.5;0.75;0.75;1), *Y*_5_
*=* (0.75;1;1;1).

To perform the defuzzification, the centre of gravity (COG) method is used [29]. Therefore, it should be mentioned, that for practical reasons, to avoid extra scaling in the computer processing, we use some extended fuzzy sets. For maximal torque it is *Y*_1_ = (−0.5, 0, 0, 0.5) and *Y*_3_ = (0.5, 1, 1, 1.5), to make it possible to obtain exactly 0 and exactly 1 as defuzzified boundary values. The same extension is used for the boundaries of the relative torque fuzzy sets. In fact, all variables in presented solution follow that pattern.

In the maximal torque module, we have an interpretation which identifies non-monotonical behavior. Low and high values should have a lower evaluation, but the middle values should have a higher result. For that, we use the rules:

*R*_1_*: IF X_R_^MT^ is^low^X_R_^MT^ANDX_L_^MT^ is^low^X_L_^MT^THENe^MT^ = Y*_1_, *R*_2_*: IF X_R_^MT^ is^low^X_R_^MT^ AND X_L_^MT^ is^med^X_L_^MT^ THEN e*^MT^* = Y*_2_, *R*_3_*: IF X_R_^MT^ is^low^X_R_^MT^ AND X_L_^MT^ is^high^X_L_^MT^ THEN e^MT^ = Y*_1_, *R*_4_*: IF X_R_^MT^ is^med^X_R_^MT^ AND X_L_^MT^ is^low^X_L_^MT^ THEN e^MT^ = Y*_2_, *R*_5_*: IF X_R_^MT^ is^med^X_R_^MT^ AND X_L_^MT^ is^med^X_L_^MT^ THEN e^MT^ = Y*_3_, *R*_6_*: IF X_R_^MT^ is^med^X_R_^MT^ AND X_L_^MT^ is^high^X_L_^MT^ THEN e^MT^ = Y*_2_, *R*_7_*: IF X_R_^MT^ is^high^X_R_^MT^ AND X_L_^MT^ is^low^X_L_^MT^ THEN e^MT^ = Y*_1_, *R*_8_*: IF X_R_^MT^ is^high^X_R_^MT^ AND X_L_^MT^ is^med^X_L_^MT^ THEN e^MT^ = Y*_2_, *R*_9_*: IF X_R_^MT^ is^high^X_R_^MT^ AND X_L_^MT^ is^high^X_L_^MT^ THEN e^MT^ = Y*_1_*,*

where *Y*_1_, *Y*_2_, *Y*_3_ are output values for *Y^MT^* and *e^MT^* is the exit/final result of the evaluation value of the maximal torque module.

As shown, the highest output (*Y_3_*) is defined for both medium values in premises, as intended by the linguistic expectations.

For the relative torque module, we have a typical ‘monotonical’ linguistic suggestion—the higher the better. Therefore, in the rules, the higher values in the premises generate higher output values. The rules follow the pattern:

*R*_1_*: IF X_RAWY_^RT^ is^low^X_RAWY_^RT^ AND X_LAWY_^RT^is^low^X_LAWY_^RT^ANDX_RTWD_^RT^is^low^X_RTWD_^RT^ AND X_LTWD_^RT^is^low^X_LTWD_^RT^THENe^RT^ = Y*_1_, *R*_2_: *IF X_RAWY_^RT^ is^low^X_RAWY_^RT^ AND X_LAWY_^RT^ is^low^X_LAWY_^RT^ AND X_RTWD_^RT^is^low^X_RTWD_^RT^ AND X_LTWD_^RT^is^high^X_LTWD_^RT^ THEN e^RT^ = Y*_2_, *R*_3_:*IF X_RAWY_^RT^ is^low^X_RAWY_^RT^ AND X_LAWY_^RT^ is^low^X_LAWY_^RT^ AND X_RTWD_^RT^is^high^X_RTWD_^RT^ AND X_LTWD_^RT^is^low^X_LTWD_^RT^ THEN e^RT^ = Y*_2_, *R*_4_: *IF X_RAWY_^RT^ is^low^X_RAWY_^RT^ AND X_LAWY_^RT^ is^low^X_LAWY_^RT^ AND X_RTWD_^RT^is^high^X_RTWD_^RT^ AND X_LTWD_^RT^is^high^X_LTWD_^RT^ THEN e^RT^ = Y*_3_, *R*_5_: *IF X_RAWY_^RT^ is^low^X_RAWY_^RT^ AND X_LAWY_^RT^ is^high^X_LAWY_^RT^ AND X_RTWD_^RT^is^low^X_RTWD_^RT^ AND X_LTWD_^RT^is^low^X_LTWD_^RT^ THEN e^RT^ = Y*_2_, … *R*_16_: *IF X_RAWY_^RT^ is^high^X_RAWY_^RT^ AND X_LAWY_^RT^ is^high^X_LAWY_^RT^ AND X_RTWD_^RT^is^high^X_RTWD_^RT^ AND X_LTWD_^RT^is^high^X_LTWD_^RT^ THEN e^RT^ = Y*_5_*,*

where *Y*_1_*, Y*_2_, *Y*_3_*, Y*_4_*, Y*_5_ are output values for *Y^RT^* and *e^RT^* is the exit/final result of the evaluation value of the relative torque module.

The rules define that the lowest value *Y*_1_ is proper only for all ‘low’ premises and the highest value *Y*_5_ is only for all of the highest premises, as the linguistic description suggests.

The second tier is a two-module concept. As inputs, we have output values from the maximal torque and relative torque modules. Two normalized (to the [0;1] interval) variables with intuition default for the evaluation ‘the higher, the better’.

Inputs are:

*X^K60^* = {*E^MT^*,*E^RT^*}, *E^MT^*: *^low^E^MT^*= (0;0;0;1),*^high^E^MT^* = (0;1;1;1), *E^RT^*: *^low^E^RT^* = (0;0;0;1),*^high^E^RT^* = (0;1;1;1).

Output variable *Y^*K*60^*:

Y^*K*60^
*= {Y_1_*^*K*60^*; Y_2_*^*K*60^*; Y_3_*^*K*60^*}: Y_1_*^*K*60^ = (0; 0; 0; 0.5), *Y_2_*^*K*60^ = (0; 0.5; 0.5; 1), *Y_3_*^*K*60^ = (0.5; 1; 1; 1).

The rules are simply:

*R*_1_: *IF E^MT^ is ^low^E^MT^ AND E^RT^ is ^low^E^RT^ THEN e^K60^ = Y*_1_^K60^, *R*_2_: *IF E^MT^ is ^low^E^MT^ AND E^RT^ is^high^E^RT^ THEN e^K60^ = Y*_2_^K60^, *R*_3_: *IF E^MT^ is ^high^E^MT^ AND E^RT^ is ^low^E^RT^ THEN e^K60^ = Y*_2_^K60^, *R*_4_: *IF E^MT^ is ^high^E^MT^ AND E^RT^is^high^E^RT^ THEN e^K60^ = Y*_3_^K60^*,*

where *e^K^*^60^ is the exit/final result of the evaluation value of the Isokinetics 60 module.

The same structure of evaluation is implemented for the other isokinetic modules—for the 180 and 300 angular velocities.

The final tier is a three-module concept, as mentioned earlier. Here, we have normalized input data with the meaning ‘the higher, the better’. This layer aggregates the input data from the different isokinetic module evaluations.

Inputs are two-valued variables:

*E^ISOK^ =* {*E^K^*^60^*, E^K^*^180^*, E^K^*^300^}

*E^K^*^60^*: ^low^E^K^*^60^*=* (0;0;0;1),*^high^E^K^*^60^
*=* (0;1;1;1), *E^K^*^180^: *^low^E^K^*^180^ = (0;0;0;1),*^high^E^K^*^180^ = (0;1;1;1), *E^K^*^300^: *^low^E^K^*^300^ = (0;0;0;1),*^high^E^K^*^300^ = (0;1;1;1). (2)

The final output variable fuzzy sets are determined by simply dividingthe [0;1] interval to represent different possible states of input combinations. 

The output variable *O=* {*O*_1_, *O*_2_, *O*_3_, *O_4_*},consists of four fuzzy values:

*O*_1_ = (0,0,0,0.333), *O*_2_ = (0,0.333,0.333,0.667), *O*_3_ = (0.333,0.667,0.667,1), *O*_4_ = (0.667,1,1,1) (3)

The rules aggregating three input data points in the final evaluation are defined as follows:

*R*_1_: *IFE^K^*^60^*is^low^E^K^*^60^*ANDE^K^*^180^*is^low^E^K^*^180^*ANDE^K^*^300^*is^low^E^K^*^300^*THENo* = *O*_1_, *R*_2_: *IF*
*E^K^*^60^
*is*
*^high^E^K^*^60^
*AND*
*E^K^*^180^
*is*
*^low^E^K^*^180^
*AND*
*E^K^*^300^
*is*
*^low^E^K^*^300^
*THEN*
*o* = *O*_2_, *R*_3_: *IF*
*E^K^*^60^
*is*
*^low^E^K^*^60^
*AND*
*E^K^*^180^
*is*
*^high^E^K^*^180^
*AND*
*E^K^*^300^
*is*
*^low^E^K^*^300^
*THEN*
*o* = *O*_2_, *R*_4_: *IF*
*E^K^*^60^
*is*
*^low^E^K^*^60^
*AND*
*E^K^*^180^
*is*
*^low^E^K^*^180^
*AND*
*E^K^*^300^
*is*
*^high^E^K^*^300^
*THEN*
*o* = *O*_2_, *R*_5_: *IF*
*E^K^*^60^
*is*
*^high^E^K^*^60^
*AND*
*E^K^*^180^
*is*
*^high^E^K^*^180^
*AND*
*E^K^*^300^
*is*
*^low^E^K^*^300^
*THEN*
*o* = *O*_3_, *R*_6_: *IF*
*E^K^*^60^
*is*
*^low^E^K^*^60^
*AND*
*E^K^*^180^
*is*
*^high^E^K^*^180^
*AND*
*E^K^*^300^
*is*
*^high^E^K^*^300^
*THEN*
*o* = *O*_3_, *R*_7_: *IF*
*E^K^*^60^
*is*
*^high^E^K^*^60^
*AND*
*E^K^*^180^
*is*
*^low^E^K^*^180^
*AND*
*E^K^*^300^
*is*
*^high^E^K^*^300^
*THEN*
*o* = *O*_3_, *R*_8_: *IF*
*E^K^*^60^
*is*
*^high^E^K^*^60^
*AND*
*E^K^*^180^
*is*
*^high^E^K^*^180^
*AND*
*E^K^*^300^
*is*
*^high^E^K^*^300^
*THEN*
*o* = *O*_4_

The final-tier system’s purpose is the aggregation of the results from the data-source modules.

## 3. Results

The results of the study are presented in Table 3, Table 4 and Table 5.

Table 3, Table 4 and Table 5 show the normalized and aggregated values of the results for each participant in the study presented in a common range of values from 0 to 1. This is carried automatically and allows the results for a specific case to be simply related to the dynamic norm thus derived, both in the area of healthy people and, after adjusting the model, of people with deficits. The proposed model, when extending the measurements to a larger representation of the population, can serve as a tool for setting a norm.

The highest outcome median was observed in Group 3, then in Group 1, and the lowest median was observed in Group 2. This is consistent with expectations.

The calculated correlations among values from measurements and outcomes from the model were moderate to high, achieving the best values for relative torque in all studied groups (Spearman’s correlation coefficient—a non-parametric test used to measure the strength of association between two variables—from 0.576 to 0.749).

The newly developed fuzzy model allows new measurable features of the observed phenomenon to be extracted from the data, reflecting differences between populations and, ultimately, changes occurring in the parameter values. The advantage of our fuzzy-based model is the high accuracy of translating clinical procedures (diagnostic tests) into computational evaluation algorithms, while maintaining the assumptions of the linguistically described data processing model.

## 4. Discussion

The aim of the study was to build a fuzzy model of lower limb peak torque in an isokinetic mode. The secondary aim was to extract computational rules.

The analyzed values in this study are of high interest when compared with data on power and lower limb work in a vertical jump. To date, the use of artificial intelligence to build a postural stability model based on parameters extracted using the Biodex System 4 Pro has not been observed. The only study of postural control using artificial intelligence that we are aware of is the development and validation of the automatic identification of postural control patterns in children with autism spectrum disorders using a machine learning approach (naive Bayes method) [30]. These are similar to those used in the Parkinson’s disease group, where among the five supervised machine learning algorithms (logistic regression, K-nearest neighbors, naive Bayes, decision trees and random forest), the kNN method showed the best results for predicting impaired postural stability [31]. However, this did not involve athletes, nor did it use fuzzy logic, so it is difficult to compare such distant studies other than to demonstrate the usefulness of machine learning for analyzing postural control in the above-mentioned group of children [30].

The model presented in this article has demonstrated its effectiveness. It is reflected in the literature on the mechanical analysis of peak force moment and absolute force moment for the straightening and bending of the knee joint for the measured angular velocities. Concordance of the results obtained was also observed with the H/Q values [32,33,34,35]. Its proposition is assessing the lower limbs in an isokinetic mode. Additionally, it allows the observation of differences between population changes occurring in the parameter values. The advantage of the model is its accuracy and its intuitiveness in translating the clinical procedures (diagnostic tests) into computational evaluation algorithms.

The novelty of our research lies in the use of hierarchical fuzzy logic to extract computational rules from data not provided explicitly, and then determining the corresponding physiological and pathological mechanisms. The contribution of our research is to complement the ways of describing modern physiology, pathology and rehabilitation, taking into account the uncertainty, incompleteness or inconsistency of the data. Deficits in this area affect both healthy people (including athletes) and sick people: those with orthopedic deficits (after injury), as well as neurological (e.g., after stroke) and neurodegenerative (e.g., Alzheimer’s disease) deficits. Improving the efficiency of diagnosis and rehabilitation will translate into improvements in the effectiveness of maintenance of existing health and the effectiveness of rehabilitation methods and tools.

A separate problem is that both in the area of gait and balance there are no reference standards, moreover, they change over time, so there is a need to separate the so-called dynamic norm from a sample of healthy people. Computational solutions will make it easier and faster for us not only to obtain such a norm from the current population, but moreover, will allow us to compare changes in this area related to both the fashion for practicing sports and changes in the way of eating or lifestyle (transition to sedentary or remote work, i.e., with less necessary mobility during the working day, greater access to city bikes or scooters, etc.).

In the case of the analyzed group of parameters, it is also important to note that the norm is a certain range of parameters, and changes in their values can occur both upwards and downwards, but they are not equally favorable or unfavorable. Hence, there is a need to take into account the direction of changes using ordered fuzzy numbers in computational models in the future. This will allow the assessment, not only of the absolute value of the change, but also its sign, which will translate into better inference and prediction of the development of health status. Such extensive research involves the need to increase the number of patients, although the current one seems to be sufficient for the needs of the basic scope of the study. The norm identified in this study may become the nucleus of the future dynamic norm for the entire population.

### Future Research Directions

The main scientific and technical direction of further research will be the development of the described solution to Neuro-Fuzzy i.e., the combination of artificial neural networks (ANNs) and the fuzzy logic system, in which the adaptation of connections between ANN layers gives the basis for learning, and fuzzy logic gives a foothold on the concept of fuzzy sets and rules, and fuzzy reasoning [28].

The main clinical direction for further research is to refine the model to accurately reflect and predict changes, and in some cases, to build a family of models, each of which can be dedicated to a different group of conditions (with a different etiology or set of symptoms). The challenge for computational models is to increase their accuracy while reducing the time required to produce clinically relevant results. Scientists, engineers and clinicians continue to search for more efficient computational methods, techniques and algorithms, including fuzzy computing that is better suited to the specific biomedical data of healthy individuals and patients with various injuries and conditions. This is because data-driven decision-making based on machine learning (ML) can provide earlier detection and better patient care, as well as more efficient allocation of limited resources.

Looking more broadly, computational balance assessment methodology can accelerate the progress of home rehabilitation by adding an efficient and rapid quantitative assessment of balance control skills to current solutions. This will enable remote assessment, rehabilitation and care, with an individualized approach and less time and cost. This opens up a new avenue for the development of intelligent (including robotic) solutions for home rehabilitation [36].Virtual reality (VR) is a well-established technology in medicine but comprehensive studies on the possible applications of VR in posture measurement are scarce. A review of 21 studies [37] confirmed that virtual reality is often used as an additional visual stimulus in the assessment of static and dynamic posturography, with researchers particularly hopeful about VR-based postural assessment in a head-mounted display (HMD VR) [37,38].

Increased body awareness has been investigated as one of the component mechanisms of mindfulness. Two computational models were developed for each of the aforementioned cases (EC, EO) based on the postural signals of 156 healthy subjects during quiet standing under closed-eye (EC) and open-eye (EO) conditions:

- Regression model, designed to estimate each participant’s mindfulness score based on their postural cues;
- Classifier, designed to assign each participant to one of the ‘Mindful’ or ‘Non-mindful’ classes [39].

This showed that the two models designed from the EC data were more effective, which may advance further research into mindfulness [39,40].

Furthermore, the proposed algorithm may be adapted toward an application in wearable devices for athletes and elderly people (including e-shoes and e-socks).

## 5. Conclusions

The fuzzy model proposed in this article that assesses the lower limbs in an isokinetic mode has demonstrated its effectiveness. In its current form, the model provides a good starting point for further research to develop a family of artificially intelligent models for balance and postural control in healthy individuals (including those actively involved in sports) and patients with balance deficits due to disease and injury.

The proposed tool aggregates the results obtained from many measured parameters into one number. This allows for a transparent analysis of a larger number of factors that are part of the mechanisms of the studied phenomenon. Thus, the final assessment will be more accurate and complete.

## Figures and Tables

**Table 1:** Characteristics of the patients studied.

Parameter	Group 1 Deaf Soccer Players (n = 28)	Group 2 Hearing Soccer Players (n = 19)	Group 3 Deaf Untrained (n = 46)
Age [years]			
Mean	20.00	19.63	21.89
SD	3.50	1.95	4.40
Min	17	17	17
Q1	18	18	18
Median	19	19	22
Q3	21	21	25
Max	30	23	32
Body height [cm]			
Mean	175.36	172.96	179.63
SD	4.13	9.43	6.01
Min	166.80	151.30	165.00
Q1	172.60	167.10	175.60
Median	174.80	173.50	179.40
Q3	178.50	180.10	184.20
Max	184.60	191.70	190.00
Shin length [cm]			
Mean	38.55	38.45	39.56
SD	1.80	2.18	2.62
Min	33.50	35.50	34.80
Q1	37.50	36.50	38.00
Median	38.00	37.80	39.60
Q3	40.00	40.00	41.50
Max	42.00	43.10	44.50
Body mass [kg]			
Mean	70.19	66.71	73.67
SD	9.63	17.13	8.38
Min	57.70	41.60	60.10
Q1	64.40	55.80	67.90
Median	68.20	62.90	74.30
Q3	75.40	75.40	78.80
Max	92.90	103.30	94.50
Dominant leg			
L	4 (14.29%)	3 (15.79%)	7 (15.22%)
R	24 (85.71%)	16 (84.21%)	39 (84.78%)

Legend: SD—Standard Deviation, Min—Minimum, Q1—Lower Quartile, Median, Q3—Upper Quartile, Max—Maximum, L—Left, R—Right.

**Table 2:** The configuration of data intervals—base for the fuzzy system definition.

Parameter	Low Values	Medium Values	High Values	Linguistic Interpretation
Isokinetic mode 60 deg/s				
R AGON/ANTAG	0–50	45–65	60–100	the medium values are good, but low and high are bad
L AGON/ANTAG	0–50	45–65	60–100	the medium values are good, but low and high are bad
R PEAKTQ/BM AWY	0–1.2	1.0–2.4	2.3–4.5	the more, the better
L PEAKTQ/BM AWY	0–1.2	1.0–2.4	2.3–4.5	the more, the better
R PEAKTQ/BM TWD	0–0.8	0.7–1.5	1.4–3.0	the more, the better
L PEAKTQ/BM TWD	0–0.8	0.7–1.5	1.4–3.0	the more, the better
Isokinetic mode 180 deg/s				
R AGON/ANTAG	0–50	45–65	60–100	the medium values are good, but low and high are bad
L AGON/ANTAG	0–50	45–65	60–100	the medium vales are good, but low and high are bad
R PEAKTQ/BM AWY	0–0.90	0.8–1.7	1.6–3.0	the more, the better
L PEAKTQ/BM AWY	0–0.90	0.8–1.7	1.6–3.0	the more, the better
R PEAKTQ/BM TWD	0–0.7	0.6–1.3	1.2–3.0	the more, the better
L PEAKTQ/BM TWD	0–0.7	0.6–1.3	1.2–3.0	the more, the better
Isokinetic mode 300 deg/s				
R AGON/ANTAG	0–60	55–90	85–150	the medium values are good, but low and high are bad
L AGON/ANTAG	0–60	55–90	85–150	the medium values are good, but low and high are bad
R PEAKTQ/BM AWY	0–0.7	0.60–1.30	1.20–2.8	the more, the better
L PEAKTQ/BM AWY	0–0.7	0.60–1.30	1.20–2.8	the more, the better
R PEAKTQ/BM TWD	0–0.7	0.60–1.30	1.20–2.8	the more, the better
L PEAKTQ/BM TWD	0–0.7	0.60–1.30	1.20–2.8	the more, the better

**Table 3:** Fuzzy model outcomes for studied Group 1.

No. of Participant/Parameter	Value
1	0.76734109
2	0.65589328
3	0.64234552
4	0.69709596
5	0.73758406
6	0.68142442
7	0.60856813
8	0.60663099
9	0.53337415
10	0.65551133
11	0.60379564
12	0.67940767
13	0.6983585
14	0.75756501
15	0.74477273
16	0.74373664
17	0.69312008
18	0.64302757
19	0.64844364
20	0.71791784
21	0.6558216
22	0.62666147
23	0.6727858
24	0.70166569
25	0.73768627
26	0.65344389
27	0.61765752
28	0.74812219
Mean	0.67606281
SD (Standard Deviation)	0.0565327
Min	0.53337415
Q1 (Lower quartile)	0.64285706
Median	0.67609673
Q3 (Upper quartile)	0.7228344
Max	0.76734109

**Table 4:** Fuzzy model outcomes for studied Group 2.

No. of Participant/Parameter	Value
1	0.56585384
2	0.64469192
3	0.54099616
4	0.61011369
5	0.56910691
6	0.58987825
7	0.56994588
8	0.5731141
9	0.56502047
10	0.61102068
11	0.54596697
12	0.61948461
13	0.62375196
14	0.66939015
15	0.575458
16	0.58376321
17	0.59714431
18	0.59057348
19	0.58581099
Mean	0.59110977
SD (Standard Deviation)	0.03258833
Min	0.54099616
Q1 (Lower quartile)	0.5695264
Median	0.58581099
Q3 (Upper quartile)	0.61056719
Max	0.66939015

**Table 5:** Fuzzy model outcomes for studied Group 3.

No. of Participant/Parameter	Value
1	0.80400957
2	0.76927435
3	0.62282284
4	0.74328593
5	0.74373664
6	0.70207034
7	0.64074447
8	0.73227387
9	0.70003183
10	0.72986779
11	0.70734059
12	0.69860786
13	0.65737016
14	0.66614456
15	0.69351383
16	0.67523309
17	0.68521024
18	0.66333946
19	0.71704625
20	0.67574448
21	0.70851757
22	0.74564416
23	0.68427606
24	0.64150353
25	0.71149862
26	0.6788365
27	0.68999421
28	0.72515522
29	0.61814783
30	0.7283098
31	0.70268278
32	0.60633094
33	0.61681826
34	0.64672526
35	0.67162273
36	0.73704441
37	0.69031519
38	0.6860709
39	0.77480748
40	0.73948592
41	0.82803729
42	0.75687176
43	0.65334806
44	0.65969457
45	0.69795882
46	0.77084335
Mean	0.69996107
SD (Standard Deviation)	0.0490296
Min	0.60633094
Q1 (Lower quartile)	0.6675141
Median	0.69828334
Q3 (Upper quartile)	0.73167235
Max	0.82803729

## Data Availability

Not applicable.
